# 4-Phenylbutyrate (PBA) treatment reduces hyperglycemia and islet amyloid in a mouse model of type 2 diabetes and obesity

**DOI:** 10.1038/s41598-021-91311-2

**Published:** 2021-06-04

**Authors:** Sara de Pablo, Júlia Rodríguez-Comas, Daniela Díaz-Catalán, Gema Alcarraz-Vizán, Carlos Castaño, Juan Moreno-Vedia, Joel Montane, Marcelina Parrizas, Joan-Marc Servitja, Anna Novials

**Affiliations:** 1 Pathogenesis and Prevention of Diabetes Group, Institut d’Investigacions Biomèdiques August Pi i Sunyer (IDIBAPS), C. Rossello 149-153, 08036 Barcelona, Spain; 2 Centro de Investigación Biomédica en Red de Diabetes y Enfermedades Metabólicas Asociadas (CIBERDEM), Barcelona, Spain

**Keywords:** Biological models, Experimental organisms, Metabolic disorders, Homeostasis, Metabolism, Metabolic diseases, Diabetes, Metabolic syndrome, Obesity, Pre-diabetes, Biological techniques, Physiology, Diseases, Endocrinology, Molecular medicine

## Abstract

Amyloid deposits in pancreatic islets, mainly formed by human islet amyloid polypeptide (hIAPP) aggregation, have been associated with loss of β-cell mass and function, and are a pathological hallmark of type 2 diabetes (T2D). Treatment with chaperones has been associated with a decrease in endoplasmic reticulum stress leading to improved glucose metabolism. The aim of this work was to investigate whether the chemical chaperone 4-phenylbutyrate (PBA) prevents glucose metabolism abnormalities and amyloid deposition in obese agouti viable yellow (A^vy^) mice that overexpress hIAPP in β cells (A^vy^ hIAPP mice), which exhibit overt diabetes. Oral PBA treatment started at 8 weeks of age, when A^vy^ hIAPP mice already presented fasting hyperglycemia, glucose intolerance, and impaired insulin secretion. PBA treatment strongly reduced the severe hyperglycemia observed in obese A^vy^ hIAPP mice in fasting and fed conditions throughout the study. This effect was paralleled by a decrease in hyperinsulinemia. Importantly, PBA treatment reduced the prevalence and the severity of islet amyloid deposition in A^vy^ hIAPP mice. Collectively, these results show that PBA treatment elicits a marked reduction of hyperglycemia and reduces amyloid deposits in obese and diabetic mice, highlighting the potential of chaperones for T2D treatment.

## Introduction

During type 2 diabetes (T2D) evolution, β cells increase their function to compensate the peripheral insulin resistance. Over time, this situation causes β cell failure, leading to a decrease in β cell mass and function^1^. One of the hallmarks of T2D is the presence of amyloid deposits in pancreatic islets, which are associated with β cell mass reduction^2^. These deposits are mainly formed by a β cell-secreted hormone known as islet amyloid polypeptide (IAPP) or amylin. Under certain stressful settings, human IAPP (hIAPP) misfolds and aggregates forming the amyloid deposits found in the pancreas of T2D patients^3,4^ through a process known as amyloidogenesis. The process of ordered aggregation into amyloid fibrils is a common event in a broad range of cell-degenerative diseases, including Alzheimer’s Disease (AD)^5^.

A number of studies show that the toxicity of the amyloidogenic peptides lies in the oligomeric intermediates rather than in the mature fibrils. Of interest, the oligomeric intermediates of this process have been related with several toxic effects in β cells^6–8^ such as membrane disruption^9,10^, proteasome impairment^11–13^, autophagy^14–17^, endoplasmic reticulum (ER) stress^12,18,19^ or inflammation^20–22^, which may ultimately result in β cell death^2,4,6^. Thus, the conversion of a normally soluble protein into amyloid structures underlies the pathogenesis induced by hIAPP aggregation through cell toxicity and cell death^6^.

Although the primary sequence of IAPP is highly conserved throughout evolution, not all species form islet amyloid deposits^23^. In particular, human and nonhuman primates, but not rodents, express a sequence of IAPP which can form amyloid fibrils. The central region of hIAPP presents hydrophobic amino acids that are thought to be responsible for its aggregation^24^. Mouse and rat IAPP differ from the human protein mainly in this central region, with three proline residues that are absent in hIAPP, which maintains the protein soluble and prevents its aggregation^25^. Thus, mouse and rat models used for T2D research cannot recapitulate one of the hallmarks of the disease. For this reason, rodent models that stably overexpress hIAPP in β cells have been developed^26^.

Chaperones are molecules that help to correct protein folding, thus alleviating ER protein overload^27^. Chaperones have shown promising results restoring glucose homeostasis in animal models^28,29^ mainly acting towards the ER stress that characterizes T2D^18,30–32^. In this regard, chemical chaperones such as bile acid derived tauroursodeoxycholic (TUDCA) and 4-phenylbutyrate (PBA) have been shown to decrease ER stress and improve insulin sensitivity in *ob*/*ob* mice^28^. Of note, PBA has been reported to partially alleviate lipid-induced insulin resistance and β cell dysfunction in humans^33^. We have previously shown that chaperones improve the function of pancreatic INS1E-β cells overexpressing hIAPP^34,35^. Moreover, we demonstrated that oral treatment of transgenic mice overexpressing hIAPP in pancreatic β cells with the chemical chaperone PBA prevents glucose intolerance and islet inflammation^29^. This study was performed in mice until they reached 20 weeks of age. At this stage, we did not observe amyloid deposits in pancreatic islets, as longer periods of time are required to detect them^36^. Nevertheless, PBA prevented amyloid formation in hIAPP transgenic islets exposed to high glucose concentration ex vivo, most likely through a direct interaction of PBA with hIAPP oligomers^29^.

It has been demonstrated that formation of amyloid deposits is accelerated by crossing hIAPP transgenic mice with obese and insulin resistant agouti viable yellow (A^vy^) mice (A^vy^ hIAPP mice), which results in overt diabetes^37^. In order to gain insights on the therapeutic potential of chaperones for the treatment of T2D, the main objective of the present work was to test whether oral treatment with the chemical chaperone PBA could improve glucose homeostasis and prevent islet amyloid formation in obese and diabetic A^vy^ hIAPP mice.

## Materials and methods

### Transgenic mouse models and PBA treatment

Heterozygous female mice overexpressing hIAPP in β cells (FVB strain, hIAPP^Tg/0^ transgene, A/A genotype for the agouti locus, purchased from The Jackson Laboratory) were crossed with obese agouti viable yellow (A^vy^) male mice (C57BL/6J strain, A^vy^/a genotype for the agouti locus, purchased from Charles River Laboratory)^37^. The generated four-genotype offspring were named as wt (A/a), hIAPP (A/a hIAPP^Tg/0^), A^vy^ (A^vy^/A), and A^vy^ hIAPP (A^vy^/A hIAPP^Tg/0^) mice. The A^vy^ and A^vy^ hIAPP offspring were identified by their yellow coat compared to the agouti coat–colored littermates (wt and hIAPP mice)^37^. All animals were housed at the specific pathogen free (SPF) animal facility of Universitat de Barcelona, in a 12 h dark/light cycle under controlled temperature conditions. Mice were fed a regular chow diet, with free access to drinking water. All animal studies were performed in compliance with current Spanish and European legislation and with the ARRIVE guidelines, and were approved by the Ethics Committee of Universitat de Barcelona (189/18, 190/18, 192/18).

8-week-old male A^vy^ and A^vy^ hIAPP transgenic mice were left untreated (control) or treated with the chemical chaperone PBA (Scandinavian Formulas, Pennsylvania, USA) dissolved in water (1 g/kg/day) during 12 weeks^28,38^, until mice were 20 weeks old. Mice had free access to water, which was changed 3 times/week. PBA concentration in drinking water was adjusted during each change according to body weight and water intake. Body weights and random-fed and 5-h fasting glucose levels were periodically monitored during the treatment.

### Glucose tolerance test and insulin secretion

Glucose tolerance tests (GTT) were performed in mice after 15 h of fasting (8 weeks of age) or 5 h of fasting (20 weeks of age). The shorter fasting was performed in mice to reduce the stress observed in 20-week-old A^vy^ hIAPP mice when fasted overnight. Mice were intraperitoneally injected with d-glucose (1 or 2 g/kg body weight, as indicated), and glucose levels in tail vein blood samples were measured with a blood glucometer (NovaPro GLU/KET, Nova Medical) just before and 15, 30, 60 and 120 min after glucose injection. Blood samples were taken at just before and 15 min after glucose injection with Microvettes containing EDTA (Starstedt, Nümbrecht, Germany) to obtain plasma and measure insulin using an Ultra Sensitive Mouse Insulin ELISA kit (Crystal Chem, Elk Grove Village, IL, USA).

### Insulin tolerance test and HOMA-IR calculations

Insulin tolerance tests (ITT) were performed after 5 h of fasting to determine insulin resistance. Human insulin (0.75 U/Kg, Humulina Regular, Lilly, Madrid, Spain) was intraperitoneally injected, and glucose levels were measured with a blood glucometer (NovaPro GLU/KET, Nova Medical) just before and 15, 30, and 60 min after insulin injection. The homeostasis model assessment index (HOMA-IR) was calculated as follows: G_0_ × I_0_/450, where G_0_ is fasting glucose (mg/dL), and I_0_ is fasting insulin (mU/L)^39^.

### Immunohistochemistry

The pancreas was extracted from mice and fixed overnight with 4% formalin, embedded in paraffin and microsectioned. Next, pancreas sections were stained using a polyclonal guinea-pig anti-insulin antibody (Dako-Agilent Technologies, CA, USA), followed by secondary incubation with an Alexa Fluor 555 anti-guinea-pig antibody (Invitrogen, CA, USA). For amyloid staining, pancreas sections were then incubated in 0.5% Thioflavin S (Sigma-Aldrich) solution for 2 min and rinsed twice with 70% ethanol. Nuclei were stained using Hoechst 33342. Fluorescence images were obtained using Leica LAS Image Analysis software. ImageJ version 1.49 software (National Institutes of Health) was used to determine the islet area and Thioflavin S staining area. Percentage of amyloid severity related to total islet area and amyloid prevalence related to total number of islets were calculated.

### Statistical analyses

Data are expressed as mean ± SEM. To test whether the variables followed a normal distribution, D’Agostino & Pearson omnibus normality test was carried out. Statistical significance differences between two groups were determined by Unpaired Student’s t-test for parametric quantitative variables and Mann–Whitney U test for quantitative nonparametric variables. Differences among more than two groups were carried out by one-way or two-way ANOVA, followed by post hoc Tukey test. For time-course experiments, multiple global comparisons considering all the time points in each group were performed. Statistical analyses at specific time points of glucose and insulin tolerance tests were performed by using two-way ANOVA followed by Tukey’s post hoc tests, or Student’s t test when comparing only two groups. Differences were considered statistically significant when p value was lower than 0.05 (p < 0.05). Graphs and statistical analysis were carried out using GraphPad Prism software (6 version, GraphPad Software, Inc., CA, USA).

## Results

### A^vy^ hIAPP mice present early-glucose intolerance, insulin resistance and overt diabetes

To study the effects of PBA in obese mice expressing hIAPP, we crossed hIAPP transgenic mice with agouti viable yellow (A^vy^) mice, a strain of obese and insulin resistant mice. The offspring of these crosses generated four different experimental groups: wt mice (wt), hIAPP transgenic mice (hIAPP), obese mice with the A^vy^ transgene (A^vy^) and obese mice with the A^vy^ and the hIAPP transgenes (A^vy^ hIAPP).

In order to characterize the double crossing and to define the protocol for PBA administration, we first performed a temporal phenotypic characterization of the A^vy^ hIAPP model that included the assessment of glucose homeostasis and the formation of islet amyloid deposits. For this, we determined body weight, food intake, and random-fed and fasting glycemia evolution. We observed that, already at 4 weeks of age, mice carrying the A^vy^ gene presented a higher weight compared to their lean littermates that increased progressively thereafter (Supplementary Fig. S1A). The increase in weight was paralleled by increased food intake (Supplementary Fig. S1B).

**Figure 1:**
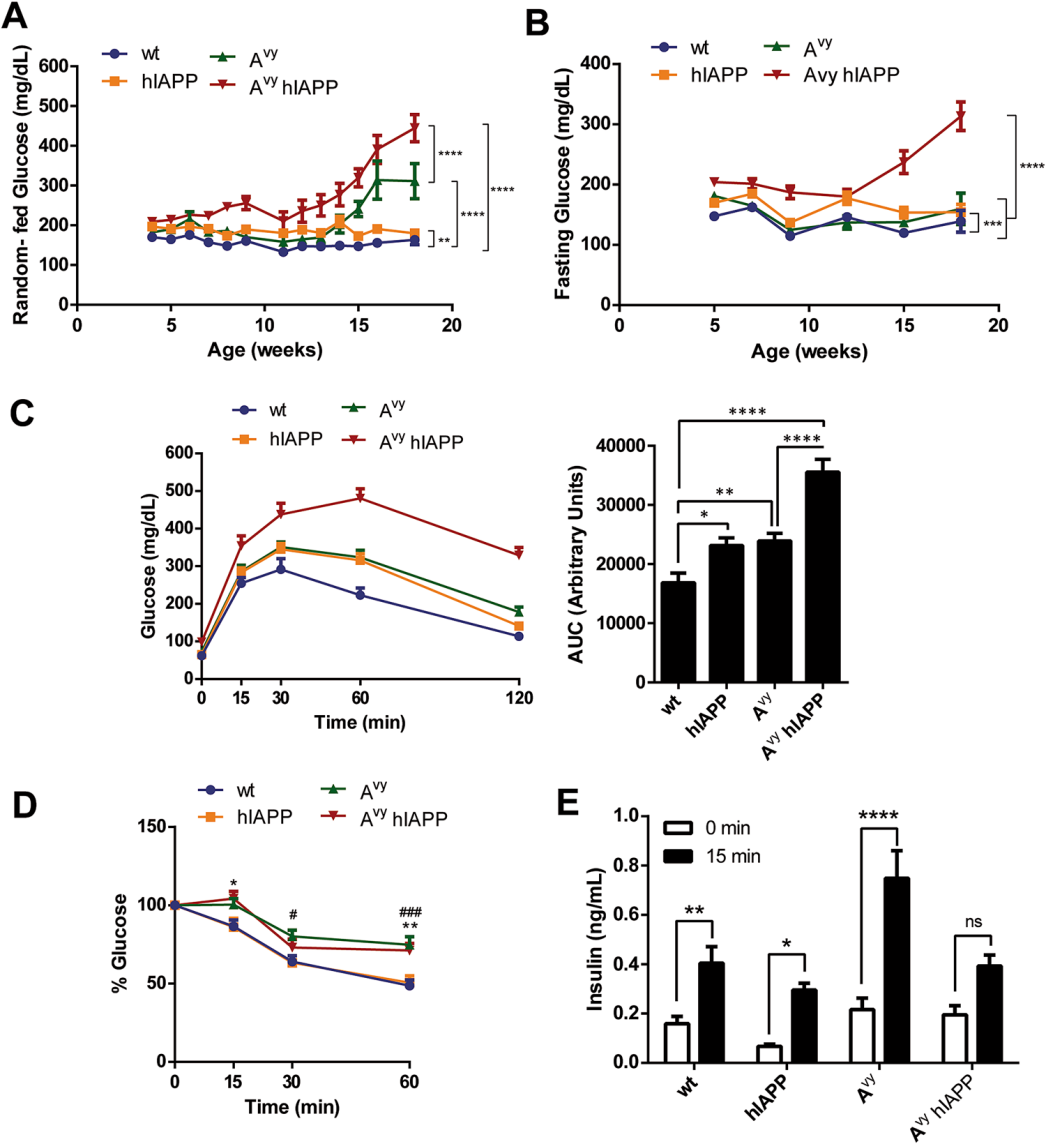
Glycemia evolution, glucose and insulin tolerance, and insulin response of A^vy^ hIAPP mice. **(A)** Fed glycemia and **(B)** 5-h fasting glycemia were evaluated in wt, hIAPP, A^vy^ and A^vy^ hIAPP mice between 4 and 18 weeks of age. (**C**) Glucose tolerance tests (2 g/kg glucose) in 8-week-old mice. Glycemia values and the respective areas under the curve (AUC) are shown. **(D)** Insulin tolerance tests (0.75 U/kg insulin) in 8-week-old mice. Percentages of glycemia values respect to the initial value are represented. **(E)** Plasma insulin levels just before and 15 min after glucose injection. Results are presented as mean ± SEM of (**A**,**B**) 7–34 determinations/group/time point, or (**C**,**D**) 12–20 and (**E**) 8–12 mice/group. Statistical analyses were performed using two-way ANOVA followed by Tukey’s post hoc tests: *p < 0.05, **p < 0.01, ***p < 0.001, ****p < 0.0001. In A and B, multiple global comparisons considering all the time points in each experimental group were performed. Statistical analysis at specific time points of the insulin tolerance test was performed by two-way ANOVA followed by Tukey’s post hoc tests: ^#^P < 0.05, ^###^P < 0.001 (A^vy^ vs. wt); *P < 0.05, **P < 0.01 (A^vy^ hIAPP vs. wt).

hIAPP mice presented mild but significant and persistent increased random-fed glycemia values compared with their wt littermates (Fig. 1A). Both A^vy^ and A^vy^ hIAPP mice showed higher fed glycemia values compared with wt mice (Fig. 1A), being the values in double transgenic A^vy^ hIAPP mice higher than in A^vy^ mice. Interestingly, random-fed glycemia started to increase earlier in A^vy^ hIAPP mice (before 10 weeks of age) than in A^vy^ mice (starting at 15 weeks, Fig. 1A). Regarding fasting glucose, no differences were observed between wt and A^vy^ mice, and only a slight increase was observed in hIAPP mice over time (Fig. 1B). In contrast, fasting glucose values were consistently elevated in A^vy^ hIAPP mice and this impairment was much more exacerbated by week 15 (Fig. 1B).

At 8 weeks of age, hIAPP and A^vy^ mice already presented glucose intolerance compared with wt mice. A^vy^ hIAPP mice were much more glucose intolerant, even compared to their A^vy^ littermates (Fig. 1C). Insulin tolerance tests revealed that both 8-week-old A^vy^ and A^vy^ hIAPP mice were similarly insulin resistant compared to their wt and hIAPP littermates (Fig. 1D), in line with their common obese phenotype. Plasma insulin levels in response to a glucose challenge were increased in A^vy^ mice in comparison to the other groups. (Fig. 1E). In contrast, the insulin response to glucose was impaired in double transgenic A^vy^ hIAPP (Fig. 1E), which is compatible with the presence of amyloid deposits is pancreatic islets, as described below.

### Time-course analysis of amyloid deposits formation in A^vy^ hIAPP mice

**Figure 2:**
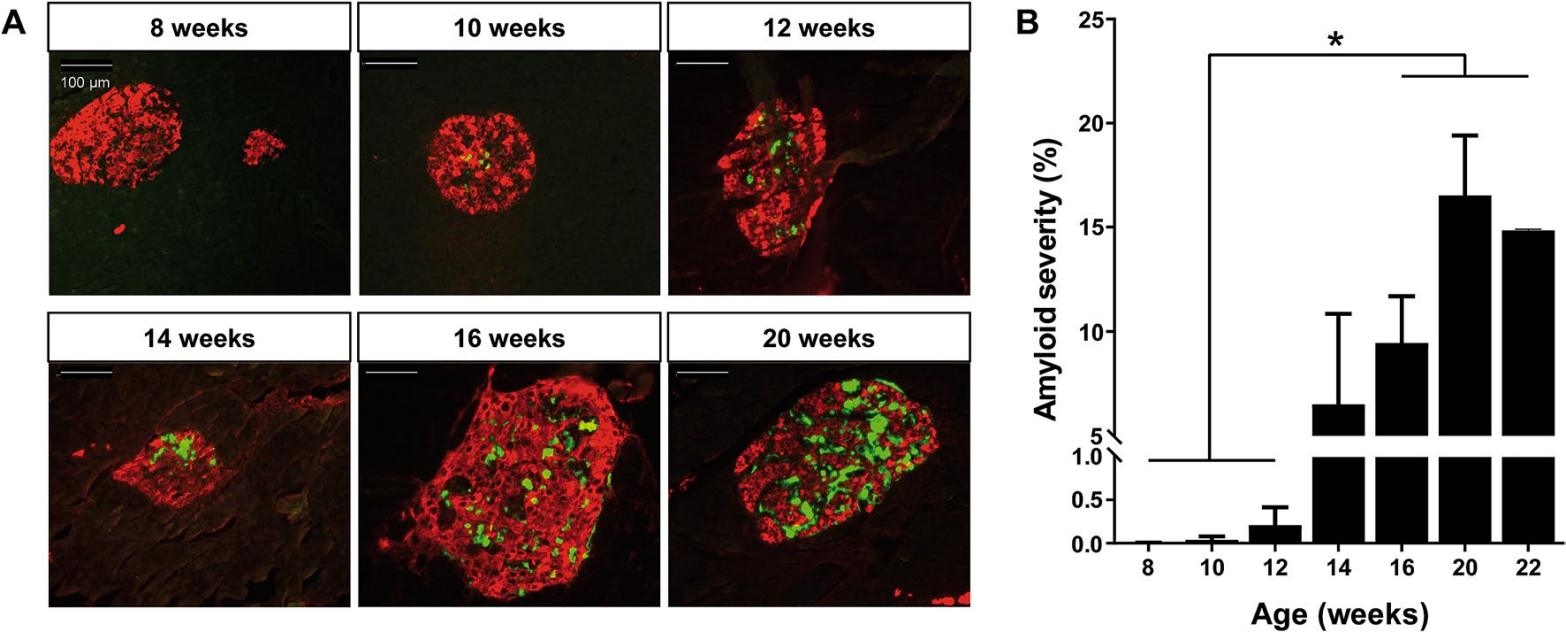
Time course of islet amyloid deposition in A^vy^ hIAPP mice. (**A**) Representative images of pancreatic islets from A^vy^ hIAPP mice at different ages. Pancreas sections were stained with Thioflavin S to study amyloid deposits (green) and an anti-insulin antibody (red). Scale bars, 100 μm. **(B)** Amyloid severity quantification (percentage of amyloid respect to total islet size). Results are presented as mean ± SEM of 2–5 mice/group. Statistical analyses were performed using one-way ANOVA. *p < 0.05.

In order to define a treatment window for PBA administration, we performed a time course study of the amyloid plaques by Thioflavin S staining in A^vy^ hIAPP mice. Amyloid deposits started to be detected at 10–12 weeks of age, presenting a slightly delayed onset with respect to the impairment of glucose homeostasis (Fig. 2A). The amyloid severity notably increased by week 14, and was stabilized around 15% at 20 weeks of age (Fig. 2B).

### PBA treatment prevents random-fed and fasting hyperglycemia in A^vy^ hIAPP mice

Once established the evolution of A^vy^ hIAPP mouse diabetic phenotype, we evaluated whether PBA treatment could prevent glucose metabolism alterations. Given that we previously reported that PBA treatment improves glucose metabolism in lean mice overexpressing hIAPP, in the present study we focused on the effect of PBA in A^vy^ and A^vy^ hIAPP mice^29^. For this, the chemical chaperone PBA was administered in drinking water (1 g/kg/day). Oral PBA treatment started at 8 weeks of age, when A^vy^ hIAPP mice already presented fasting hyperglycemia, glucose intolerance, and impaired insulin secretion. The treatment finalized at 20 weeks, when, as shown before, islet amyloid was stabilized around 15% of severity.

**Figure 3:**
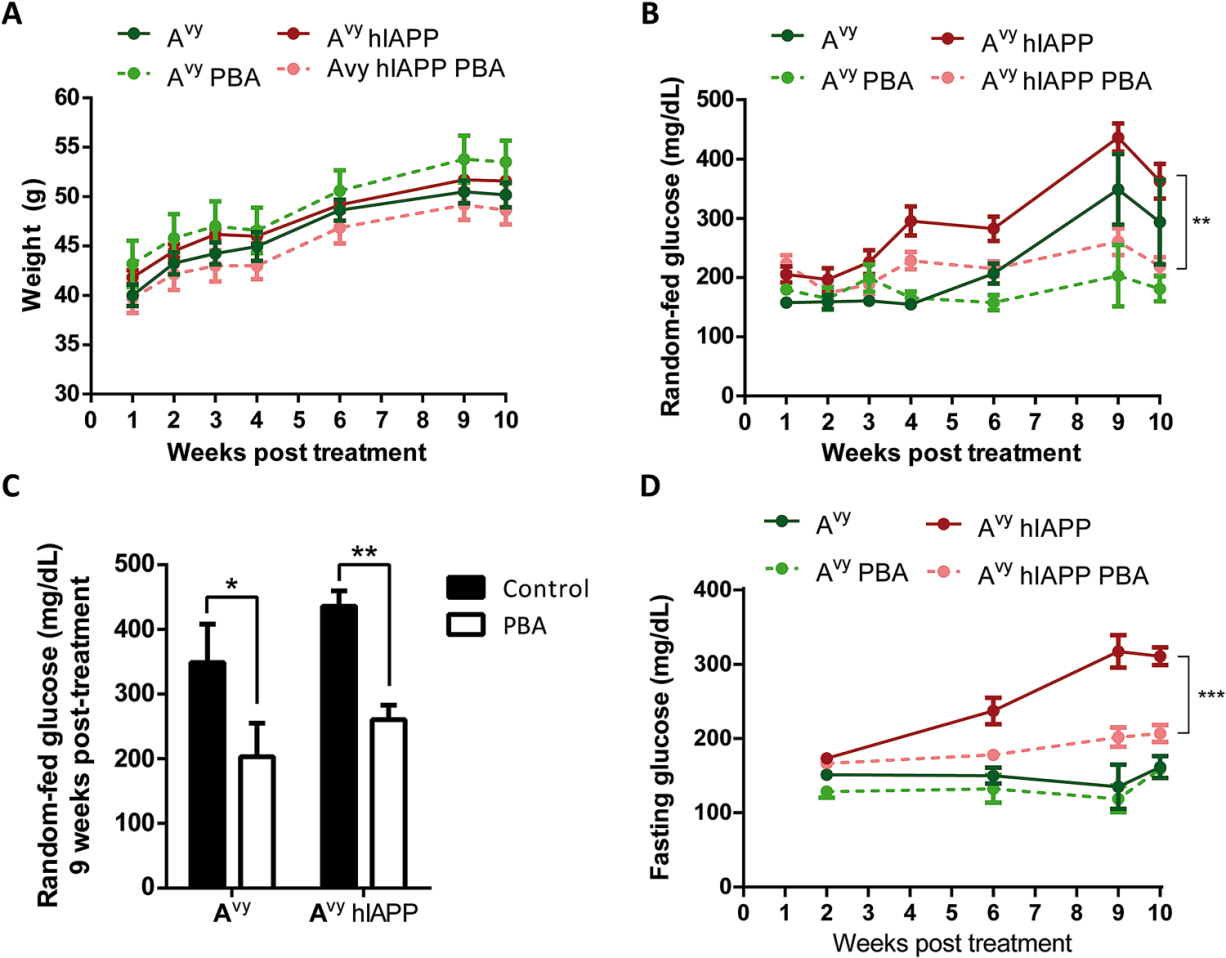
PBA treatment reduces hyperglycemia in A^vy^ hIAPP mice. (**A**) Body weight and (**B**) fed glycemia evolution of non-treated and PBA-treated mice. (**C**) Fed glucose values of 17-week-old mice, after 9 weeks of PBA treatment. (**D**) 5-h fasting glycemia evolution of non-treated and PBA-treated mice. Representative results from two independent experiments are shown. Results are presented as mean ± SEM of 4–7 mice/group. Statistical analyses were performed using two-way ANOVA followed by Tukey’s post hoc tests: *p < 0.05, **p < 0.01, ***p < 0.001. In B and D, multiple global comparisons considering all the time points in each experimental group were performed.

First, we observed that PBA treatment had no impact on body weight in A^vy^ and A^vy^ hIAPP mice (Fig. 3A). To test whether PBA had an effect on glycemia values in both groups of mice, we monitored random-fed and fasting glycemia throughout the treatment period. PBA treatment markedly reduced the hyperglycemia in fed A^vy^ hIAPP mice (Fig. 3B). The efficacy of PBA in preventing the increase of glycemia in A^vy^ hIAPP mice was observed during all the study and was more evident during the last weeks of treatment, when the values were as high as 400 mg/dL in non-treated mice and around 200 mg/dL in PBA-treated littermates (Fig. 3B,C). Remarkably, PBA treatment also induced a potent reduction of fasting glycemia, which reached very high values during the last weeks of the experiment in non-treated A^vy^ hIAPP mice (Fig. 3D). A^vy^ mice did not show increased fasting glycemia values throughout the study (Fig. 3D), and only presented increased random-fed glycemia during the last weeks of the treatment, which was also prevented by PBA treatment (Fig. 3B,C).

### PBA treatment in A^vy^ hIAPP mice ameliorates glucose intolerance and hyperinsulinemia

**Figure 4:**
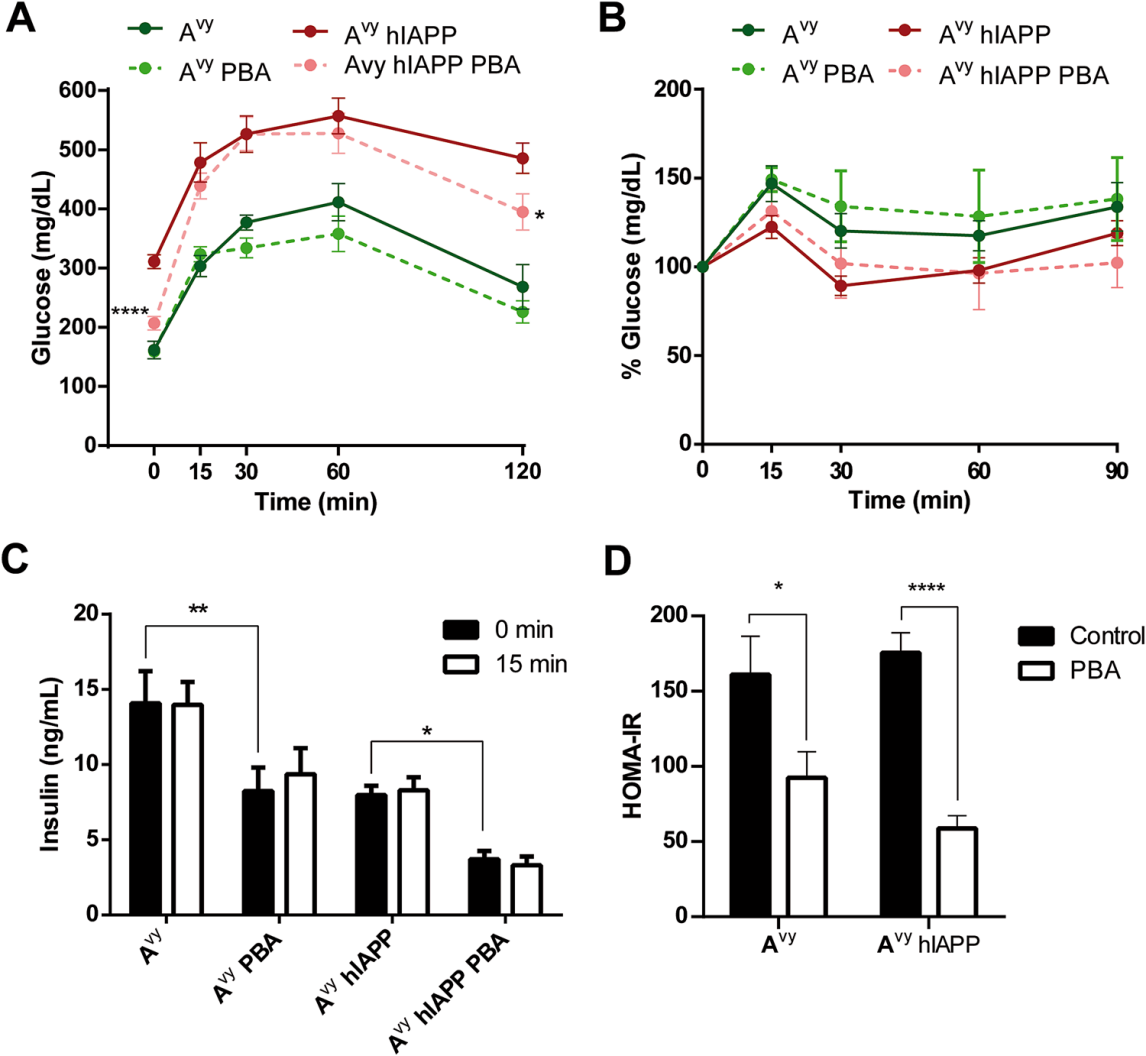
PBA treatment improves glucose homeostasis in A^vy^ hIAPP mice. **(A)** Glucose tolerance tests (1 g/kg glucose) in 18-week-old A^vy^ and A^vy^ hIAPP mice treated or not with PBA for 10 weeks. (**B**) Insulin tolerance tests (0.75 U/kg insulin). Percentages of glycemia values respect to the initial value are represented. **(C)** Plasma insulin levels after a 5-h fasting (0 min) or 15 min after glucose injection (15 min). **(D)** HOMA-IR index was calculated using 5-h fasting glucose and insulin levels. Results are presented as mean ± SEM of 4–7 mice/group. Statistical analyses were performed by using (A) Student’s t test at each time point comparing each treated group vs. the respective non-treated control group, or (C,D) two-way ANOVA followed by Tukey’s post hoc tests: *p < 0.05, **p < 0.01, ****p < 0.0001.

Glucose metabolism was further studied by glucose tolerance tests 10 weeks after the initiation of PBA treatment, when mice were 18 weeks old (Fig. 4). Globally, PBA did not significantly improve glucose tolerance in A^vy^ and A^vy^ hIAPP mice (Fig. 4A). However, it should be noted that the analysis of the glucose tolerance tests was hampered by a technical underestimation of glycemia in A^vy^ hIAPP mice as many values reached the detection limit of the glucometer (600 mg/dL). Indeed, 60 min after glucose administration, glycemia in 50% of non-treated A^vy^ hIAPP mice reached the detection limit, whereas only 16% of the PBA-treated A^vy^ hIAPP mice presented such values, indicating that the glycemic values were higher and over range in non-treated mice compared to their PBA-treated littermates (Fig. 4A). In line with this, after 120 min of glucose administration, glycemia values were significantly lower in treated A^vy^ hIAPP mice (Fig. 4A). Together with the lower basal glucose levels observed in treated A^vy^ hIAPP mice, these results demonstrate that PBA treatment improves glucose homeostasis in these mice.

We next evaluated the insulin sensitivity by insulin tolerance tests (ITTs) at 18 weeks of age. Both A^vy^ and A^vy^ hIAPP mice presented a potent insulin resistance that was not improved by PBA (Fig. 4B). This lack of effect of PBA may be due to a severe insulin resistance, too high to detect differences by a conventional dose of insulin when performing the ITT. Indeed, the fasting insulin levels observed in all A^vy^ groups were very high (reaching values as high as 15 ng/mL in A^vy^ mice), which is consistent with a severe insulin resistance state (Fig. 4C). Importantly, PBA treatment did reduce the fasting insulin levels in A^vy^ and A^vy^ hIAPP mice, illustrating a partial amelioration of insulin resistance that was not uncovered by the ITTs (Fig. 4C). However, we did not observe an increase in insulin levels in any of the four experimental groups after a glucose challenge, indicating that the islet response to glucose remained blunted even in PBA-treated mice (Fig. 4C).

Taken advantage of the fasting glycemia and insulinemia values, we calculated HOMA-IR, as an index of insulin resistance. Of note, HOMA-IR was decreased in both A^vy^ and A^vy^ hIAPP mice treated with PBA compared with their non-treated controls to a similar extent (Fig. 4D), supporting again that PBA treatment reduces insulin resistance in both experimental groups. Thus, mice treated with PBA were able to improve (A^vy^ hIAPP) or maintain (A^vy^) glycemic values with lower insulin levels respect to non-treated littermates.

### PBA treatment in A^vy^ hIAPP mice reduced islet amyloid deposition

**Figure 5:**
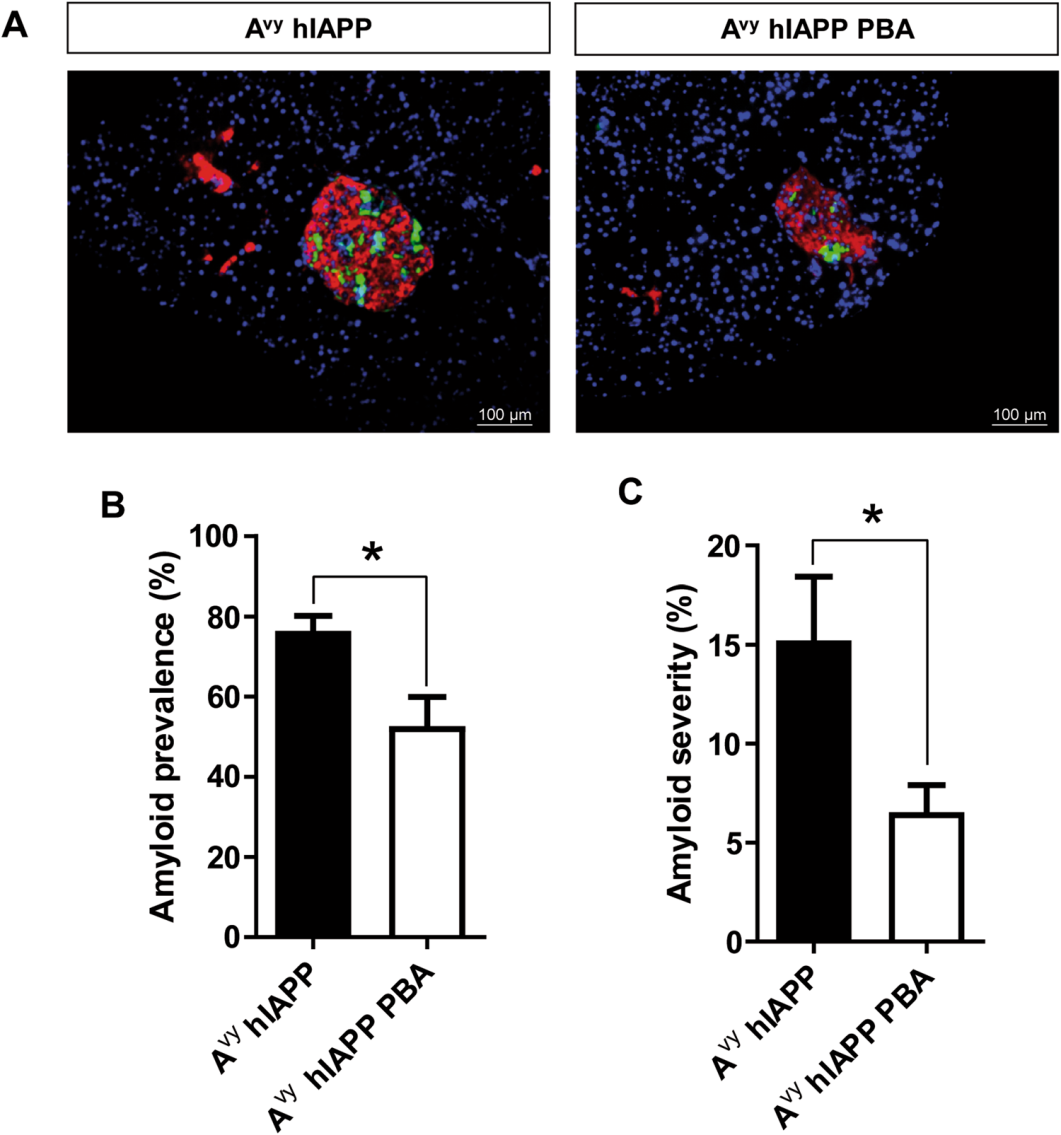
PBA treatment prevents amyloid formation in A^vy^ hIAPP mice. **(A)** Representative images of islets from 20-week-old A^vy^ hIAPP mice after 12 weeks of treatment with oral PBA. Non-treated mice were used as controls. Pancreas sections were stained with Thioflavin S to detect amyloid deposits (green), an anti-insulin antibody (red), and Hoechst 33,342 (blue). Scale bars, 100 μm. **(B)** Amyloid prevalence (% of islets with amyloid) and **(C)** amyloid severity (% of amyloid to total islet size). Results are presented as mean ± SEM of 7–8 mice/group, from 2 independent experiments. Statistical analyses were performed using Student’s t-test. *p < 0.05.

Finally, we aimed to determine whether PBA treatment had an impact on the deposition of amyloid deposits in A^vy^ hIAPP mice. For this, pancreas sections from 20-week-old A^vy^ hIAPP mice and after 12 weeks of treatment were stained with Thioflavin S (Fig. 5A). PBA treatment decreased amyloid prevalence, calculated as the percentage of pancreatic islets with a positive Thioflavin S staining (Fig. 5B). Remarkably, amyloid severity was reduced by almost 60% in PBA-treated A^vy^ hIAPP mice compared with non-treated littermates (Fig. 5C).

## Discussion

Patients with T2D are characterized by increased glycemic values due to insulin resistance and/or β-cell dysfunction. One of the hallmarks of T2D is the presence of amyloid deposits in pancreatic islets^2,4^, formed by the β cell-secreted peptide hIAPP^40^. Chaperone treatment has emerged as a potential therapeutic approach for T2D. In the present study, we show that oral treatment with the chemical chaperone PBA improves glucose homeostasis and reduces amyloid deposits in a mouse model characterized by obesity and β cell dysfunction due to hIAPP overexpression, highlighting the potential of this drug for the treatment of T2D.

The so called group of amyloidogenic diseases, including Alzheimer’s disease (AD), Parkinson disease, some rare diseases, and T2D, share the accumulation of abnormally folded and insoluble proteins that interfere with cell function^41^. While in AD brain amyloid is formed by the aggregation of Aβ protein, in T2D islet amyloid is formed by aggregation of hIAPP, a peptide hormone co-secreted with insulin by β cells in response to glucose. hIAPP misfolds into oligomers, which results in amyloid plaque formation. hIAPP aggregation is toxic for β cells, leading to β cell death^2,4,12^. Thus, preventing amyloid formation is a logical approach in drug discovery for T2D, AD and other amyloidogenic diseases.

We previously reported that PBA treatment of lean mice overexpressing hIAPP in β cells ameliorates glucose intolerance^29^. This study was performed in mice until they reached 20 weeks of age, when increased basal glucose levels and islet amyloid deposits in pancreatic islets are still not detected. In the present study, we have used the A^vy^ hIAPP mice model, generated by crossing hIAPP transgenic mice with obese and insulin resistant A^vy^ mice. This cross has been shown to induce obesity, insulin resistance, overt diabetes and large islet amyloid deposits^37^. In order to characterize the appropriate treatment window to evaluate the efficacy of PBA in vivo, we first performed a weekly analysis of the random-fed and fasting glucose levels in blood. We observed that A^vy^ hIAPP consistently presented increased random-fed glycemia earlier than A^vy^ mice, reaching values as high as 400 ng/mL at 18 weeks of age. Moreover, A^vy^ hIAPP also presented increased fasting glycemia at very early stages and dramatically increased from 12 to 18 weeks of age. In parallel, we performed an extensive time-course evaluation of the formation of amyloid deposits, since other studies have performed analyses in more advances stages or in specific time points^37,42^. Amyloid deposits started to appear in A^vy^ hIAPP mice approximately at 10–12 weeks of age and increased over time. At 18–20 weeks of age, the amyloid severity was stabilized at 15%. Therefore, according to our characterization of the model, we decided to implement an oral PBA treatment in A^vy^ hIAPP mice starting at 8 weeks of age and finalizing at 20 weeks of age in order to evaluate the efficacy of this drug against both hyperglycemia and islet amyloid formation.

Our results demonstrate that PBA treatment potently reduced random-fed glycemia and fasting hyperglycemia observed in A^vy^ hIAPP mice. PBA was also able to decrease random-fed glycemia in obese A^vy^ mice. Remarkably, the capacity of PBA to prevent increased glycemia was accompanied by a reduction of the hyperinsulinemia observed in both A^vy^ and A^vy^ hIAPP mice. Therefore, lower insulin levels were needed to maintain similar or even lower glycemic values in PBA-treated mice, which is illustrated by the reduced HOMA-IR values observed in PBA-treated A^vy^ and A^vy^ hIAPP mice. Although the HOMA-IR index was created using large clinical studies in humans, multiple studies in rodents have shown a correlation between this index and measures based on hyperinsulinemic euglycaemic clamps^43,44^, the gold standard for insulin resistance determination. HOMA-IR has been indeed used as a reasonable and reliable surrogate for IR in many studies performed in mice^45^. We could not detect an improvement of insulin sensitivity by a conventional insulin tolerance test, most likely due to a severe insulin resistance in A^vy^ and A^vy^ hIAPP, as shown by the severe hyperinsulinemia observed in these mice at 20 weeks of age. For instance, the basal insulin levels were as high as 15 ng/mL and 8 ng/mL in 20-week-old A^vy^ and A^vy^ hIAPP mice, respectively, compared to 0.2 ng/mL in the same mice at 8 weeks of age.

The reduced hyperinsulinemia observed in PBA-treated mice is in line with the reported insulin-sensitizing effects of chemical chaperones, including PBA, on peripheral tissues. For instance, Özcan et al. demonstrated in *ob/ob* mice that treatment with TUDCA and PBA decreased ER stress and ameliorated hyperglycemia by improving insulin sensitivity in white adipose tissue and liver^28^. PBA has also been related with reduction of body weight through decreasing adipogenesis in epididymal white adipose tissue^46^ and sensitizing for leptin signaling in hypothalamus^30^. It is important to note that in our study, PBA improved glucose metabolism in obese A^vy^ and A^vy^ hIAPP mice by decreasing glycemia and basal hyperinsulinemia without affecting body weight, which is more in accordance to the results by Özcan et al.^28^.

Remarkably, the beneficial effects of PBA on glucose homeostasis were paralleled by a decrease in islet amyloid deposition in A^vy^ hIAPP mice. These results are in line with our previous studies showing that PBA reduces amyloid formation in transgenic hIAPP islets cultured at high glucose concentration^29^ and other studies showing that chaperones inhibit amyloid deposition^34,38,47,48^. Our previous studies pointed to a direct interaction between PBA and hIAPP oligomers and fibrils, ensuring a preventive and even a reversing effect on amyloid aggregation, that was paralleled by a restoration of glucose homeostasis in lean hIAPP mice^29^. Since in A^vy^ hIAPP mice amyloid formation is triggered by the insulin resistance of these mice, it is very difficult to ascertain whether the improvement in glycemia and amyloid is due to the PBA effect to improve insulin sensitivity or improve β cell function, or both. The fact that PBA reduces HOMA-IR in both A^vy^ and A^vy^ hIAPP, and that the blunted glucose-stimulated insulin secretion in these mice is not alleviated by PBA, suggests that this chaperone may act mainly through the improvement of insulin sensitivity, although we cannot rule out that the effect on amyloid deposition may be potentiated by a direct effect on pancreatic islets. Thus, we can speculate that a reduced β cell overload illustrated by reduced hyperinsulinemia, together with the direct anti-amyloidogenic effects of PBA, would generate a double beneficial effect of this drug in reducing amyloid deposition in pancreatic islets.

Other agents have been shown to protect β cells against hIAPP-induced toxicity. For instance, targeting islet inflammation with an IL-1 receptor antagonist improved glucose tolerance in obese hIAPP transgenic mice, although amyloid accumulation was not reduced^42^. Similarly, we recently demonstrated that the anti-inflammatory molecule alpha1-antitrypsin ameliorated glucose intolerance and β cell dysfunction in hIAPP transgenic mice^49^. Moreover, we also showed that overexpression of the chaperone protein disulfide isomerase (PDI), which does not play a direct role in ER stress, restored insulin secretion in hIAPP transgenic islets without reducing amyloid deposition^35^. PBA treatment has the advantage that can be administered orally, has a reduced cost, presents a long-term safety profile, and has been approved by the U.S. Food and Drug Administration (FDA) for clinical use in urea-cycle disorders and cystic fibrosis^50,51^. In this regard, PBA has been reported to partially alleviate lipid-induced insulin resistance and β cell dysfunction in humans^33^. This, together with the fact that PBA is able to act on the peripheral tissues and prevent and even reverse amyloid deposits, provides a superior beneficial effect of PBA with respect to other agents that have also been shown to successfully protect hIAPP transgenic islets.

In conclusion, our results show that treatment with oral PBA improves glucose homeostasis and prevents amyloid deposition in A^vy^ mice that overexpress hIAPP, an obese mouse model with amyloid-induced islet dysfunction causing severe hyperglycemia. These results highlight the therapeutic potential of chemical chaperones for the treatment of T2D.

## Supplementary Information

Supplementary Information.

## Abbreviations

A^vy^: Agouti viable yellow
ER: Endoplasmic reticulum
GTT: Glucose tolerance test
HOMA: Homeostatic model assessment
hIAPP: Human islet amyloid polypeptide
IAPP: Islet amyloid polypeptide
ITT: Insulin tolerance test
PBA: 4-Phenylbutyrate
TUDCA: Tauroursodeoxycholic acid
T2D: Type 2 diabetes
Wt: Wild type
