# Supplementary material for: 4-Phenylbutyrate (PBA) treatment reduces hyperglycemia and islet amyloid in a mouse model of type 2 diabetes and obesity
Source: Sci Rep. 2021 Jun 4;11:11878. doi: 10.1038/s41598-021-91311-2 (PMC8178353; doi:10.1038/s41598-021-91311-2)
Supplement: Supplementary file 1 — Supplementary Information. [file 41598_2021_91311_MOESM1_ESM.pdf]

Supplementary Information

4-Phenylbutyrate (PBA) treatment reduces hyperglycemia and islet amyloid in a mouse model of type 2 diabetes and obesity

Sara de Pablo[1], Júlia Rodríguez-Comas[1], Daniela Díaz-Catalán[1,2], Gema Alcarraz-Vizán[1,2], Carlos Castaño[1,2], Juan Moreno-Vedia[1], Joel Montane[1], Marcelina Parrizas[1,2], Joan-Marc Servitja[1,2,#,*], Anna Novials[1,2,#,*]

[1] Pathogenesis and prevention of diabetes group, Institut d'Investigacions Biomèdiques August Pi i Sunyer (IDIBAPS), Spain. [2] Centro de Investigación Biomédica en Red de Diabetes y Enfermedades Metabólicas Asociadas (CIBERDEM), Spain.

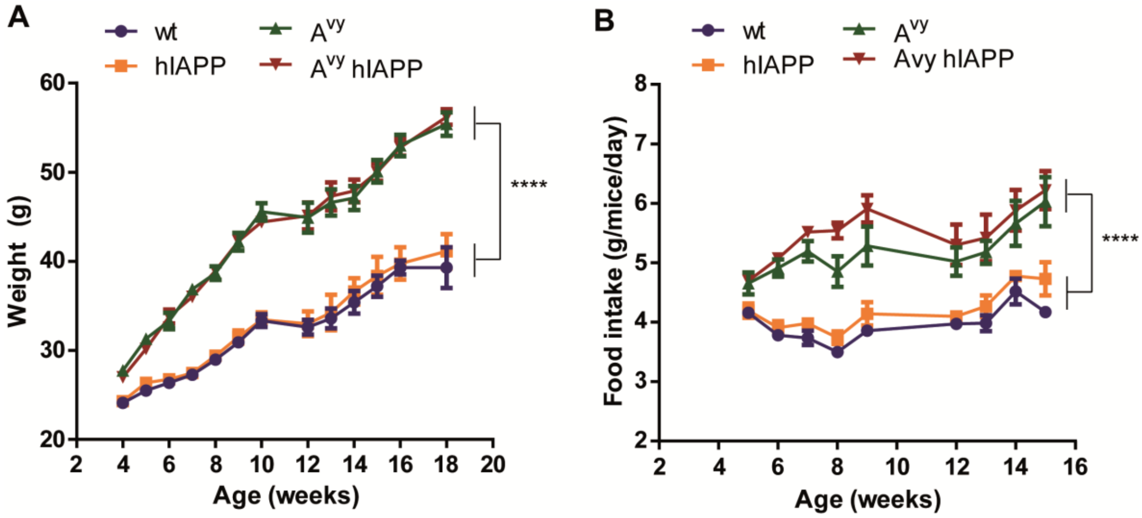

Supplementary Figure 1. Body weight and food intake evolution of A^{vy} hIAPP mice. (A) Body weight and **(B)** food intake were evaluated in wt, hIAPP, A^{vy} and A^{vy} hIAPP mice between 4 and 18 weeks of age. Results are presented as mean ± SEM of (A) 7-34 mice/group or (B) 2-8 cage measurement/group. Multiple global comparisons considering all the time points in each experimental group were performed using two-way ANOVA followed by Tukey's post hoc tests: ****p<0.0001.
